# New acoustic monitoring system quantifying aspiration risk during monitored anaesthesia care

**DOI:** 10.1038/s41598-023-46561-7

**Published:** 2023-11-18

**Authors:** Yoshitaka Shimizu, Shinichiro Ohshimo, Noboru Saeki, Kana Oue, Utaka Sasaki, Serika Imamura, Hisanobu Kamio, Eiji Imado, Takuma Sadamori, Yasuo M. Tsutsumi, Nobuaki Shime

**Affiliations:** 1 Department of Dental Anesthesiology, Graduate School of Biomedical and Health Sciences, Hiroshima University, 1-2-3 Kasumi, Minami-Ku, Hiroshima, 734-8553 Japan; 2 Department of Emergency and Critical Care Medicine, Graduate School of Biomedical and Health Sciences, Hiroshima University, 1-2-3 Kasumi, Minami-ku, Hiroshima, 734-8551 Japan; 3 Department of Anesthesiology and Critical Care, Graduate School of Biomedical and Health Sciences, Hiroshima University, 1-2-3 Kasumi, Minami-ku, Hiroshima, 734-8551 Japan; 4 Department of Dental Anesthesiology, Division of Oral & Maxillofacial Surgery and Oral Medicine, Hiroshima University Hospital, 1-2-3 Kasumi, Minami-ku, Hiroshima, 734-8553 Japan

**Keywords:** Medical research, Translational research

## Abstract

Respiratory monitoring is crucial during monitored anaesthesia care (MAC) to ensure patient safety. Patients undergoing procedures like gastrointestinal endoscopy and dental interventions under MAC have a heightened risk of aspiration. Despite the risks, no current system or device can evaluate aspiration risk. This study presents a novel acoustic monitoring system designed to detect fluid retention in the upper airway during MAC. We conducted a prospective observational study with 60 participants undergoing dental treatment under MAC. We utilized a prototype acoustic monitoring system to assess fluid retention in the upper airway by analysing inspiratory sounds. Water was introduced intraorally in participants to simulate fluid retention; artificial intelligence (AI) analysed respiratory sounds pre and post-injection. We also compared respiratory sounds pre-treatment and during coughing events. Coughing was observed in 14 patients during MAC, and 31 instances of apnoea were detected by capnography. However, 27 of these cases had breath sounds. Notably, with intraoral water injection, the Stridor Quantitative Value (STQV) significantly increased; furthermore, the STQV was substantially higher immediately post-coughing in patients who coughed during MAC. In summary, the innovative acoustic monitoring system using AI provides accurate evaluations of fluid retention in the upper airway, offering potential to mitigate aspiration risks during MAC.

Clinical trial number: jRCTs 062220054.

## Introduction

Monitored anaesthesia care (MAC) requires various respiratory monitoring devices to ensure patient safety and optimize anaesthesia delivery. However, certain procedures under anaesthesia, such as gastrointestinal endoscopy and dental procedures, pose a high risk of aspiration due to the overlap between the airway and the operative field^1–3^. Guidelines for MAC recommend respiratory monitoring using capnography, which measures the concentration of carbon dioxide in exhaled breath and provides continuous monitoring of the patient's ventilation^4,5^. Capnography can also detect changes in respiratory rate and depth, as well as the presence of airway obstruction. However, capnography cannot detect upper airway water retention, and measurements using nasal gas collection can be affected by various factors, including exhaled gas dilution from nasal cannulas used for oxygen administration, high nasal flow, and breathing pattern^6–10^. During MAC, anaesthetics such as propofol and benzodiazepine agonists, can reduce the salivary swallow reflex by decreasing peripheral muscle activity. Additionally, mechanical stimulation during the procedure can result in excessive salivary retention in the upper airway, necessitating the anaesthesiologist’s constant vigilance to prevent aspiration^11–13^.

To prevent aspiration during MAC, it is crucial to detect salivary retention early and take appropriate measures, such as adjusting head position, suctioning saliva, and reducing the dose of anaesthetics. However, diagnosing aspiration through classical auscultation is subjective and requires skill, making it impractical for anaesthesiologist’s to constantly use a stethoscope intraoperatively^14–16^. To address this issue, the present study developed a novel acoustic monitoring system that objectively and visually evaluates breath sounds. The system displays respiratory sounds as a spectrogram in real time^17^, and uses an algorithm to analyse irregular breath sounds. Furthermore, artificial intelligence (AI) analysis of inspiratory inflow sounds acquired at the neck enables quantification of the level of water retention in the upper airway^18^.

This study aimed to confirm the clinical feasibility of an AI acoustic analysis system for detecting fluid retention in the upper airway, and to determine whether respiratory monitoring combined with capnography can quantify aspiration risk.

## Methods

### Ethical approval

This was a prospective observational study conducted at Hiroshima University Hospital, which was approved by the Certified Review Board of Hiroshima University (approval number: CRB2022-0002). The trial was registered prior to patient enrolment at the Japan Registry of clinical trials (jRCTs062220054, Principal investigator: Yoshitaka Shimizu, link to trial registry, Date of registration: August 31, 2022; https://jrct.niph.go.jp/en-latest-detail/jRCTs062220054). All methods were performed in accordance with the relevant guideline and regulations, including Strengthening the Reporting of Observational Studies in Epidemiology (STROBE) guidelines^19^. Written informed consent was obtained from all participants, who could withdraw at any time.

### Participants

Inclusion criteria were: ≧18 years of age, capability to undergo dental procedures on an outpatient basis, dental phobia, abnormal (excessive) gag reflex, and undergoing invasive dental procedures. Exclusion criteria were: allergy and a history of serious adverse reactions to midazolam or propofol, dysphagia, poor oral hygiene, and mental disability. Sixty-seven patients, who underwent supervised anaesthesia management at Hiroshima University Hospital between September and November 2022, were initially enrolled in this study. However, five participants who did not consent to the study and two who had anaesthesiologist protocol errors were excluded.

### Anaesthesia and data collection

**Figure 1:**
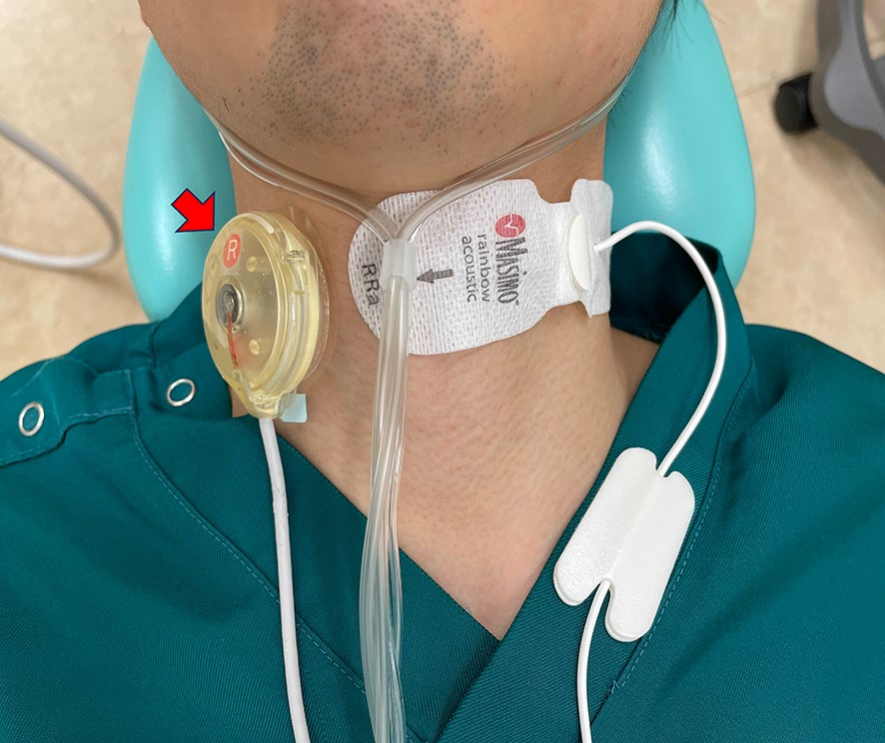
The cervical sensor is placed just lateral to the thyroid cartilage and medial to the sternocleidomastoid muscle (on the right side, indicated by a red arrow). The RRa sensor is attached contralaterally (on the left side).

**Figure 2:**
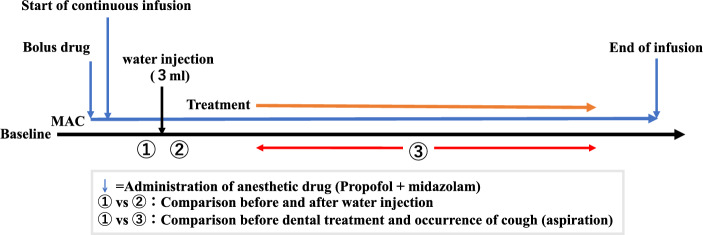
Anaesthesia and data collection protocol diagram. STQV assessments were performed in a quiet environment to avoid noise effects. *MAC* monitored anaesthesia care, *STQV* stridor quantitative value.

The CARESCAPETM B850 bedside monitor (GE Healthcare, Chicago, IL, USA) was used to measure blood pressure, SPO_2_, electrocardiogram, and capnogram. Subsequently, the venous route was secured, and propofol (10 or 20 mg bolus, followed by 0.8–4 mg/kg/h as continuous dosage) and midazolam (1–2.5 mg bolus) were administered. Sedation levels were managed within the range of 3–4 on the Observer's Assessment of Alertness/Sedation (OAA/S) scale^20^. An acoustic respiratory rate (RRa) sensor of the Radical 7 monitor (Masimo Corp., Irvine, CA, USA) was attached to the skin of the left paralarynx using adhesive tape, whereas the sensor of the continuous monitoring system for respiratory sounds was attached to the skin of the right paralarynx. (Fig. 1). Oxygen was administered at 3 L/min through a nasal cannula. Subsequently, in accordance with the recommendations for the swallowing evaluation drinking test, 3 cc of water was injected into the oral cavity using a syringe, and the stridor quantitative value (STQV) was compared before and after the injection^21^. Dental treatment was then initiated, and STQV was noted when coughing (aspiration) occurred during dental treatment. The quantitative waveforms in each respiratory monitoring device were confirmed using the respiratory sound continuous monitoring system when no respiration was detected for > 20 s, according to the capnogram and RRa. (Fig. 2). Sampling of all subjects was performed in the supine position. The capnogram in the capnography, the audiogram in the RRa, and the spectrogram in the new respiratory sound monitoring system were observed. Electronic data used in this study was from an electronic data capture system (REDCap™).

### Respiratory sound monitoring system and calculation of the STQV

**Figure 3:**
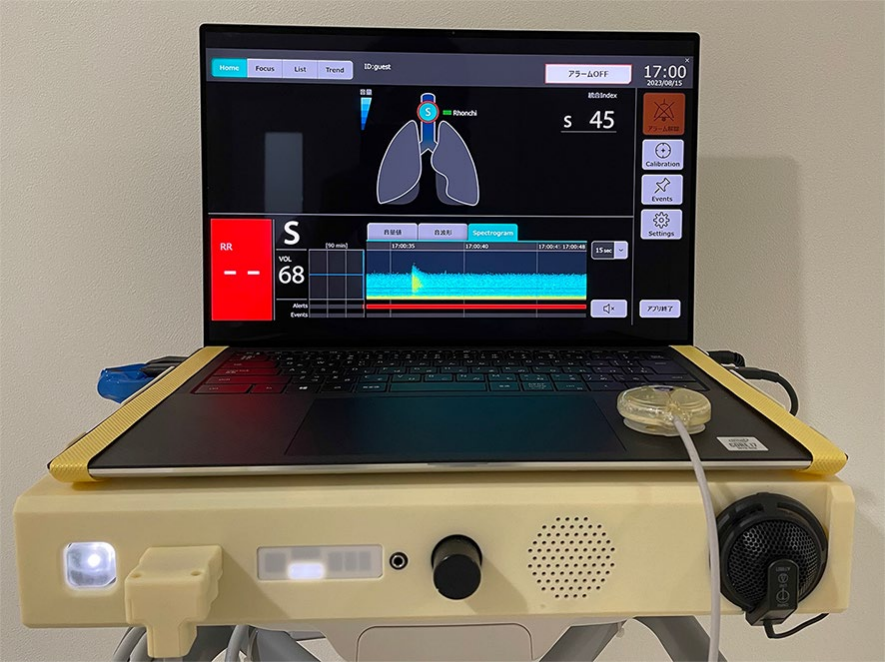
The respiratory sound monitoring system comprises sensors for detecting respiratory sounds and the PC for visualization.

The respiratory sound monitoring system consists of a sensor for breath sound detection, a control unit (prototype) and a laptop computer (DESKTOP-H94L8BJ, Dell Technoligies Inc, Round Rock, TX, USA), and the AI acoustic analysis algorithm for calculating STQV is embedded in a dedicated installed application. (Fig. 3). The STQV was calculated in this study as a quantitative measure of mucus retention using cervical auscultation. An AI analysis algorithm was developed to calculate the STQV, using 56 labelled sounds as the training dataset for machine learning and 35 labelled sounds as the validation dataset. Feature parameters were extracted from snoring, caused by upper airway obstruction because of subsidence of the tongue root, and from stridor sounds, generated by the resonance of water-soluble reservoirs between inspiration and expiration. This extraction utilised frequency analysis, cepstrum analysis, the Liftering process, and other methods.

**Figure 4:**
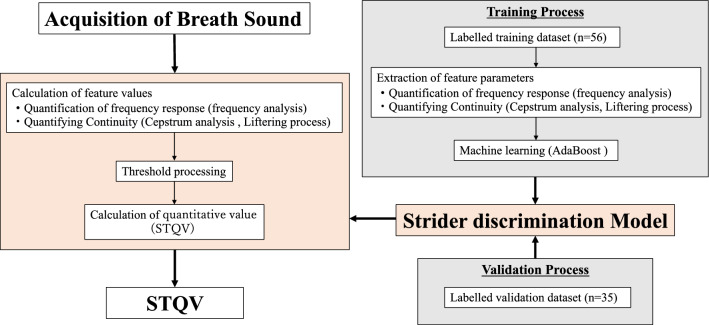
Machine learning algorithms for the values of the strider component. Based on a training dataset of strider sounds recorded from patients and labelled by experts, features were extracted using frequency and cepstrum analyses, and machine learning algorithms were applied.

Features are calculated after every extremely short time period (< 100 ms, fixed). If a feature exceeds the threshold determined by machine learning, the period is identified as an "abnormal sound period," and if it does not exceed the threshold, it is identified as a "non-abnormal sound period." The larger the feature, especially when the abnormal sound is loud, the higher is the probability of it being in the "abnormal sound period". A quantitative value (range, 0–1) of abnormal respiratory sounds can be obtained by calculating the ratio between the number of "abnormal sound periods" and the total number of sections (Fig. 4).

### Sample size and statistical analysis

In this pilot study, it was not possible to obtain abnormal detection data based on STQV in cervical auscultation, so the sample size estimation was based on a previous study by the same research group that used fine crackles sound AI analysis for interstitial lung disease detection. This previous study showed that the fine crackles sound AI index was 0.032 ± 0.023 and 0.121 ± 0.090 in the control and interstitial lung disease group, respectively^17^, from which an effect size of 0.95 was calculated using the G*Power software (version 3.1.9.6). The final target sample size was estimated as 17 participants using an alpha level of 0.05 and a power of 0.95. However, assuming a 30% incidence of aspiration in MAC, the target number of participants needed was 51. Assuming a participation rate of 0.8, the final target number of patients was set at 60.

SPSS (version 25 for Mac, IBM, Inc, Chicago, IL, USA) was used for statistical analyses, and statistical significance was set at P < 0.05. The STQVs were analysed using a paired t-test.

## Results

Sixty participants (35 women and 25 men) were included in this study, with a mean age of 47.9 ± 15 [min–max: 20–84] years, a mean height of 160.2 ± 9.5 [min–max: 140–183] cm, and a mean weight of 60.6 ± 16.3 [min–max: 38–118] kg. Patients with American Society of Anesthesiologists Physical Status Classification 1 or 2 were included. The mean anaesthetic doses of propofol and midazolam used for MAC were 100 ± 44 [min–max: 37–240] mg and 2.2 ± 0.6 [min–max: 1–5] mg, respectively, and the mean anaesthetic duration was 50.4 ± 16.5 [min–max: 21–97] min.

**Figure 5:**
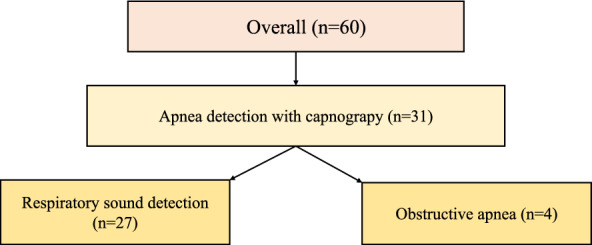
Breakdown of individuals with apnoea detected by capnogram. Respiratory sounds were confirmed using audiograms and spectrograms.

During the MAC procedure, 14 participants developed cough. Apnoea was detected by capnogram during expiratory nasal sampling in 31 cases (52%), of which 27 cases had breath sounds (Fig. 5).

**Figure 6:**
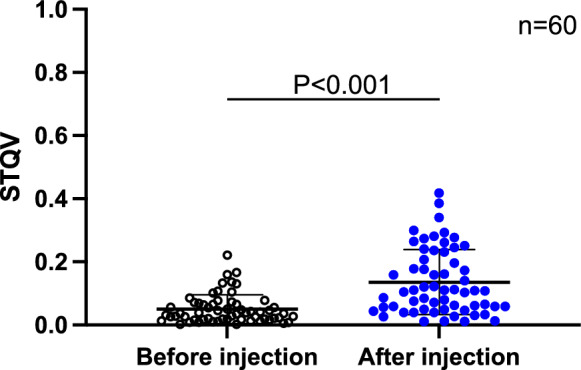
Comparison of the values of STQV before and after intraoral injection of 3 cc of water. The STQV was significantly higher after intraoral water injection than that before (0.136 ± 0.103 vs. 0.05 ± 0.045, P < 0.001 [95% confidence interval (CI) − 0.11 to − 0.061]. The horizontal line indicates the mean, and error bar indicates the standard deviation; analysis was performed using the student’s t-test. *STQV* stridor quantitative value.

**Figure 7:**
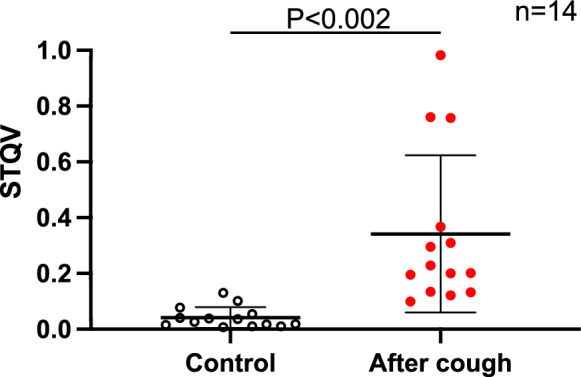
Comparison of the STQV in patients with cough. The STQV pre-treatment and immediately after the onset of coughing in patients who developed cough during MAC (n = 14) was 0.042 ± 0.04 and 0.342 ± 0.038, respectively, indicating that the STQV was significantly higher immediately after the onset of coughing (P < 0.002 [95% CI − 0.47 to − 0.13]. The horizontal line indicates the mean, and error bar indicate standard deviation; analysis was performed using the student’s t-test. *STQV* stridor quantitative value.

The results of the stridor quantitative value (STQV) analysis based on AI were as follows. The STQV was significantly higher after intraoral water injection than that before (0.136 ± 0.103 vs. 0.05 ± 0.045, P < 0.001 [95% confidence interval (CI); –0.11 to –0.061]; Fig. 6). The STQV pre-treatment and immediately after the onset of coughing in patients who developed cough during MAC (n = 14) was 0.042 ± 0.04 and 0.342 ± 0.038, respectively, indicating that the STQV was significantly higher immediately after the onset of coughing (P < 0.002 [95% CI − 0.47 to − 0.13]; Fig. 7).

## Discussion

In this study, capnography with nasal expiratory gas sampling was effective in detecting apnoea. Notably, of the 31 patients (52%) who had apnoea detected using capnography during MAC, 27 (87%) had breath sounds detected, and obstructive apnoea was present in < 13%. Our results suggest that capnography can detect mouth breathing under MAC.

The STQV, as analysed by AI, increased during the dental procedure due to fluid retention in the upper airway, caused by water injection and bleeding. It was particularly increased following the coughing event.

While the risk of aspiration during MAC can be minimized through preoperative fasting, parasympathetic nerve blockers, and other techniques, such as head elevation and proper patient positioning, it cannot be completely eliminated in all cases. Therefore, patients receiving MAC should be closely monitored for signs of aspiration throughout the perioperative period^1–4^.

Capnography is a device that can continuously monitor exhaled carbon dioxide concentration and is useful in detecting changes in respiratory rate, respiratory depth, and the presence of airway obstruction. However, it cannot detect aspiration. Moreover, if the sampling tube for capnography is implanted only the nose, measuring capnogram is impossible in mouth breathing^6–10^. There is a correlation between mouth breathing and aspiration in patients with sleep apnoea^22,23^. Therefore, this information may be useful to identify patients at risk of aspiration.

The respiratory centre increases alveolar ventilation to regulate breathing during sedation, when there is an acute partial obstruction of the upper airway resulting in symptoms, such as snoring. Therefore, when a partial obstruction causes a decrease in alveolar ventilation, the respiratory centre senses it and prolongs the inspiratory duty cycle (the time spent inhaling relative to the entire respiratory cycle) to stabilize ventilation^24–26^. However, the prolonged inspiratory duty cycle in the mouth-breathing state further increases the risk of aspiration.

In such a mouth-breathing state, if the level of fluid retention in the upper airway of patients receiving MAC could be accurately determined, the risk of aspiration could be reduced using capnography. The present study’s research group has developed smart stethoscopes that utilize machine learning algorithms to analyse tones and provide real-time analysis results. One of the valuation scales developed, called the STQV, was designed to identify excessive mucus accumulation in the upper airway using its acoustic technology. In the study, the STQV was found to increase in patients who received MAC due to the infusion of water into the upper airway, which is known to increase with salivation, suppression of swallowing function by anaesthetics, and mechanical stimulation by devices inserted through the mouth such as bite blocks. In the present study, intentional intervention (intraoral water injection) increased water retention in the upper airway and STQV, suggesting that the effect was triggered by a fluid retention in the upper airway.

The STQV may not be the most effective method for evaluating aspiration risks because liquid in the larynx may not necessarily produce distinctive sounds that can be detected using acoustic sound monitoring. Nonetheless, the acoustic sound analysis technology using AI analysis can detect increased water retention in the upper airway.

The RRa of an existing respiratory sound monitoring device can detect breath depression (a decrease in breathing rate) before it becomes severe, helping to prevent respiratory failure and the need for invasive care. However, there is little advantage in using the existing device in combination with capnograms, as their measurement points overlap^27–31^.

Acoustic monitoring using AI analysis technology has the potential to diagnose various pathological conditions, such as wheezing and blistering sounds. The present study confirmed its usefulness in assessing upper airway presence of fluid. In the future, the diagnostic capabilities of this system could be expanded by confirming the location of airway reservoirs and analysing voice patterns^17,18,32,33^.

This study had some limitations that should be considered when interpreting the results. Firstly, the criteria for sedation management, such as the anaesthetics dosage and sedation level, were not clearly defined in advance and were left to each anaesthesiologist’s discretion. This may have led to variability in the level of sedation and thus influenced the study's findings. Secondly, the STQV values calculated in this study were absolute values and were not calibrated for each patient, which may have contributed to variations in the measurements. Thirdly, this study only included patients undergoing dental treatment who are exposed to a high risk of aspiration^33^, which therefore, limits the generalizability of the findings to other types of procedures with different aspiration risks. Fourth, the condition of the upper airway fluid reservoir, caused by intraoral water injection performed as an intervention, has different properties from those of saliva or blood; the noises generated may also exhibit different tones. Fifth, the STQV readings can be affected by the noise generated by dental instruments, it was not possible to confirm the exact STQV immediately before this event. It is plausible that it was even higher prior to the coughing than after it. Finally, future research should expand the application of the proposed technique to other medical procedures and examine its validity in a randomized controlled trial.

In summary, the results of this study confirm that an AI-powered respiratory sound diagnostic system can assess upper airway fluid retention in the non-intubated state, suggesting its usefulness for evaluating the risk of aspiration during MAC.

## Data Availability

The data supporting this study’s findings are available from the corresponding author upon reasonable request.
